# *Helicobacter pylori* infection progresses proximally associated with pyloric metaplasia in age-dependent tendency: a cross-sectional study

**DOI:** 10.1186/s12876-018-0883-y

**Published:** 2018-10-29

**Authors:** Huiying Shi, Hanhua Xiong, Wei Qian, Rong Lin

**Affiliations:** Department of Gastroenterology, Union Hospital, Tongji Medical College, Huazhong University of Science and Technology, 1277 Jiefang Avenue, Wuhan 430022, China

**Keywords:** Helicobacter pylori, Pyloric glands, Metaplasia, Aged

## Abstract

**Background:**

The elderly population presents higher morbidity of *H. pylori* associated diseases in proximal stomach*.* The specific pathogenesis and mechanism have not been clearly addressed. The gastric environment for *H. pylori* colonization is dynamic with increasing age. The aim of present study is to investigate the correlation among the distribution of *H. pylori*, mucosal inflammation, gastric microenvironment and age.

**Methods:**

A total of 180 patients with dyspepsia symptoms were divided into young, middle-aged and elderly groups. Biopsies were obtained from each patient in five locations: great curvature (mid-corpus, mid-antrum), lesser curvature (mid-corpus, mid-antrum) and incisura angularis (IA), analyzed for *H. pylori* density, mucosal inflammation and histopathology.

**Results:**

The infection rate of *H. pylori* increased linearly with age (*p* <  0.001) in corpus, but not in antrum and IA. The *H. pylori* density was significantly aggravated in IA (*p* = 0.002) and corpus (*p* <  0.001) in elderly patient, but not in antrum. The mucosa inflammation scores were consistent with the severity of *H. pylori* colonization among three age groups. In elderly patients, the pyloric glands present more frequently in corpus, comparing with young and middle-aged group. A significant positive correlation among aggravating severity of *H. pylori* infection, mucosal inflammation and pyloric metaplasia in corpus with increasing age (*p* <  0.001) was occurred.

**Conclusions:**

With increasing age, both topographic distribution of *H. pylori* and the expansion of pyloric glands increased in a distal-to-proximal gastric direction. Pyloric metaplasia in corpus was correlated with the risk of aggravated *H. pylori* colonization and associated inflammation in elderly population.

## Background

Helicobacter pylori were catapulted to the hot field of gastroenterological research in less than three decades after its discovery in 1983 by Warren and Marshall [1]. Since then, evidence has accumulated to link *H. pylori* to chronic gastritis, gastric ulcer, gastric carcinoma and lymphoma [2–10], which are referred to as *H. pylori* associated diseases. Eradication of *H. pylori* significantly decreases the risk of these diseases.

There is convincing evidence that the elderly population has a significantly higher mean morbidity of *H. pylori* associated diseases than young people [11–13]. Graham et al. have reported that *H. pylori* infection prevalence increased gradually with age, leveling out at 60–70% in elderly people from less than 20% in 25–30-year-olds [14]. Note, *H. pylori* associated diseases, such as ulcers and gastric cancer, are more evenly distributed throughout the stomach in the elderly, particularly in proximal stomach [11, 12, 15]. However, the specific pathogenesis and mechanism of the high prevalence of *H. pylori* associated diseases in proximal stomach of elderly population have not been clearly addressed.

*H. pylori* organisms within the mucous layer are exposed to different local microenviorment in the antrum and body. *H. pylori* have been shown to present a strong affinity to gastric-type epithelium, prefer to colonize first and initiate in the lower part of stomach (antrum) [15, 16]. Within different gastric niches, the pH levels [17], distribution and viscosities of mucin glycoforms [18] and binding of *H. pylori* to gastric mucins in a pH-dependent manner [19] might impact on the *H. pylori* adaption*.* Recent studies have reported that gastric mucin plays dual roles in preventing gastric cancer by inhibiting *H. pylori* infection and suppressing tumor-promoting inflammation [20–22].

At the meanwhile, the tendency of pyloric gland moving to proximal stomach changed with increasing age has been demonstrated [15, 17]. In gastric body or at the body-antrum junction, the gland was specially replaced by mucous-secreting glands with increasing age, which is identified as pyloric metaplasia or named as antralization. Therefore, our hypothesis was that age-related pyloric metaplasia can effect *H. pylori* distribution in stomach, which might contribute to the high morbidity of *H. pylori* associated disease in the elderly population, especially in proximal stomach. The dynamic characteristics of *H*.*pylori* colonization in different gland types with increasing age has not been reported yet.

Our study aims to investigate the correlation among age, *H. pylori* distribution, gastric gland type and mucosa inflammation in different areas of stomach.

## Methods

### Patients

A total of 180 patients (105 men, median age: 45.5 years, range: 20–80 years) who were admitted to outpatient clinic center with complains of dyspepsia and present the normal findings of upper gastrointestinal endoscopy were enrolled into this study. This study was conducted at Wuhan Union Hospital. Exclusion criteria were: (1) patients with visible abnormalities detected through endoscopy; (2) previous *H. pylori* eradication treatment; (3) use of PPI (proton pump inhibitors), H2-receptor antagonists, bismuth preparations and antibiotics in the preceding two weeks; (4) severe or unstable cardiovascular, pulmonary, renal or hepatic disease or endocrine disease in whom endoscopy would not be safe; (5) hematological disorder or concomitant anticoagulant; (6) pregnant or nursing women.

Patients were divided into three groups: young group (20–25 years old), middle-aged group (40–50 years old) and elderly group (> 60 years old). There were 60 patients in each group. Informed consent was obtained from all patients.

### Endoscopy procedure

We employed both white light endoscopy using an OLympus GIF-Q260Z instrument, and endoscope-based confocal laser endomicroscopy (CLE) using a Cellvizio GastroFlex UHD instrument. A full assessment of the upper gastrointestinal tract for each patient was carried out first using white light endoscopy according to a standard protocol. One endoscopist (R.L.) experienced with endomicroscopy carried out the CLE. At least 10 images were obtained from the great curvature, lesser curvature and incisura angularis (IA). *H. pylori* infection was diagnosed on the basis of CLE criteria while the image was generated as described in previous study [23].

**Fig. 1:**
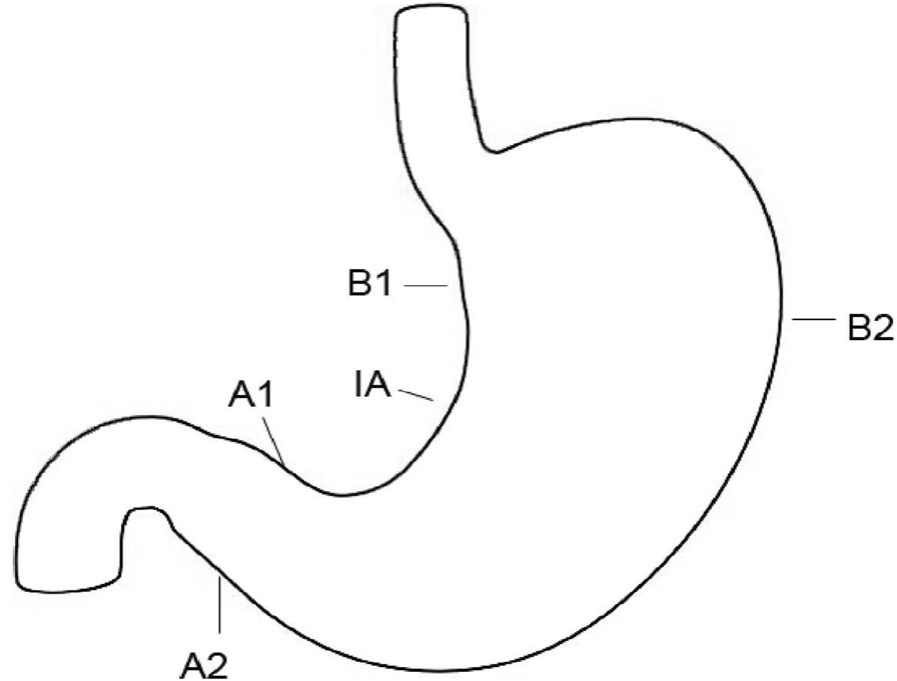
A schematic illustration of Gastric biopsies carried out in present study. Gastric biopsies were obtained from the stomach in five locations: lesser curvature (mid-antrum, A1; mid-corpus, B1), greater curvature (mid-antrum, A2; mid-corpus, B2) and incisura angularis(IA) according to the updated Sydney system

In each patient, biopsies from 5 predetermined sites: great and lesser curvature of the mid-antrum (2-3 cm from pylori, A1, A2), lesser curvature of the mid-body (4 cm proximal from IA, B1), great curvature of the mid-body (8 cm from cardia, B2) and IA, as described in updated Sydney system were taken [2, 24] (Fig. 1).

### Histology and *H. pylori* status

Each biopsy was immediately subjected to a 1-min ultra-rapid urease test (URUT test kit, Sanqiang Bio. co., Jiangsu, China), and the color change was noted after 1 min. Then formalin fixation and paraffin blocks were prepared. The sections stained with haematoxylin and eosin for histopathological details were used to demonstrate gland type and mucosa inflammation. The Giemsa staining and CLE images was performed to measure *H. pylori* density.

*H. pylori* infection was confirmed when URUT and histopathological result were both positive. The *H. pylori* density in single field was grades as: 0, normal (did not detect *H. pylori* colonization, *Grade 0*); 1, sporadic (single cluster was detected in one villus, *Grade 1*); 2, small amount (*H. pylori* diffused distributed in more than one villus, *Grade 2*); 3, moderate (the medium amount of *H. pylori* in the field, *Grade 3*); 4, marked (the field was filled with *H. pylori*, *Grade 4*). And the mucosal inflammation was scored as: 0, normal; 1, very mild; 2, mild; 3, moderate; 3, marked [24]. The *H. pylori* infection in antrum was defined as positive with either A1 or A2 present positive. The average score in A1 and A2 was used to evaluate *H. pylori* infection and mucosal inflammation in antrum for each patient. So did B1 and B2 in corpus.

### Statistical analysis

The *H.pylori* infection rates and detection rates of pyloric gland in antrum, IA and corpus with increasing age were analyzed by a Chi-square test, respectively. The density of *H.pylori* colonization and *H. pylori* associated mucosal inflammation in antrum, IA and corpus with increasing age were tested by Kruskal-Wallis H test, respectively. The density of *H.pylori* colonization between young group and elderly group was tested by One-way ANOVA. A chi-square test was performed to analyze the correction between the mucosal inflammation and *H. pylori* colonization. The Pearson correlation was used to observe the relation between *H. pylori* infection rate and proximal expansion of pyloric gland. The correlation between the colonization of *H. pylori*-associated mucosal inflammation and the gland type expansion was determined with a chi-square test. The correlation among *H. pylori* colonization, severity of mucosal inflammation, pyloric metaplasia and age was further measured with zero-order (Pearson) multiple correlations. Statistical significance of differences and relationships were determined by *p* values of less than 0.05.

## Results

### *H. pylori* infection rates increase with age in the gastric corpus

**Fig. 2:**
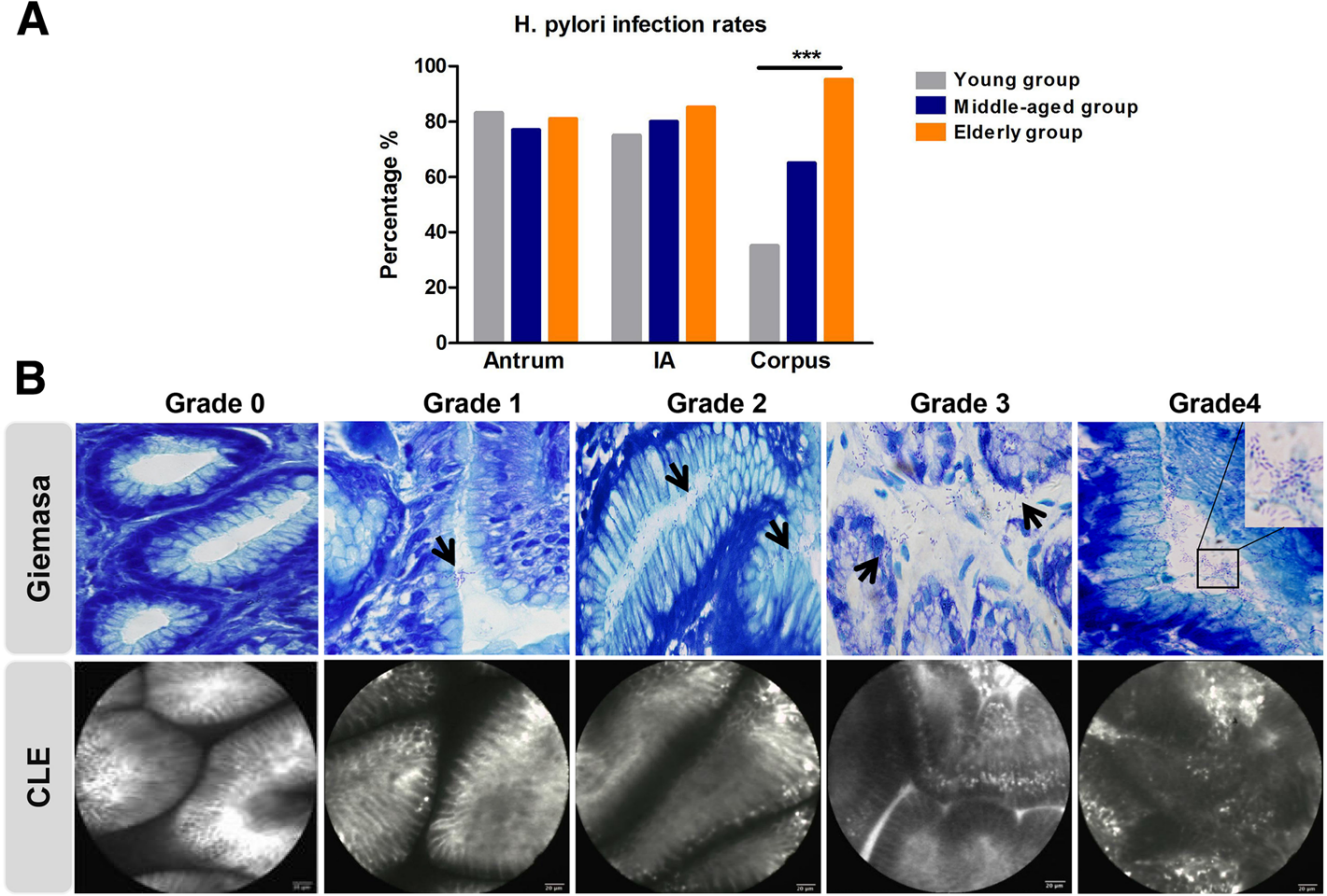
The distribution and severity of *H. pylori* in stomach in different age groups. **a** *H. pylori* infection rates in antrum, incisura angularis (IA) and corpus with increasing age. **b** The *H. pylori* colonization density was graded by Giemsa staining (upper panel) and confocal laser endomicroscopy (lower panel). A total of 20 high power (× 40 objective) microscopic fields were randomly choosed in each Giemsa staining sample, and the average scores of those 20 fields for each slide were defined as *H. pylori* density scores. *** *p* < 0.001

*H. pylori* infection rates were 83%, 77% and 81% respectively in three age-based groups, (young, middle-aged, elderly group) in antrum. The rates presented 75%, 80% and 85% in IA (Fig. 2a*)*, and there was no significance among three groups. The infection rates in corpus increased linearly with age, being 35%, 65%, and 95% in young, middle-aged and elderly populations separately (Fig. 2a, *p* <  0.001). The data indicated that *H. pylori* infection rate presented as an age-dependent tendency in corpus, but not in antrum and IA.

### *H. pylori* colonization extend proximally with increasing age

The severity of *H. pylori* colonization were further detected by Giemsa staining and CLE examination as described in methods (shown in Fig. 2b). The mean *H. pylori* density scores in antrum were around 1.5 without significant difference among three age groups (Table 1). The *H. pylori* density scores were dramatically elevated in IA (*p*=0.031) and corpus (*p* <  0.001) comparing among young, middle-aged and elderly group. With increasing age, there was no statistically difference of *H. pylori* grading/density score in antrum, the scores in IA were increasing slightly (the difference present between young group and elderly group, but not middle-aged group), while the elevation of infection scores in corpus was significant. It suggests that *H. pylori* distribution in proximal atomach augmented with increasing age.

**Table 1:** The severity and distribution of H.pylori colonization with increasing age

Age group	Antrum	IA	Corpus	*P* Value
Young group (20y-25y)	1.30 ± 0.11	1.12 ± 0.11*	0.50 ± 0.10	< 0.001
Mid-aged group (40y-50y)	1.52 ± 0.14	1.25 ± 0.12	1.05 ± 0.12	0.031
Elderly group (>60y)	1.58 ± 0.13	1.55 ± 0.12*	1.58 ± 0.10	0.974
Kruskal-Wallis H (p)	0.257	0.031	< 0.001	–

*means a statistically significant difference between young group and elderly group in IA, *p* = 0.002. *IA* incisura angularis

### *H. pylori* associated mucosal inflammation aggravate in elderly patient, especially in proximal stomach

Among three age groups, the mean grading/density scores for mucosal inflammation were 1.20, 1.57, and 1.72 respectively in antrum, 0.85, 1.45, 1.77 in IA and 0.37, 1.03, 1.83 in corpus (Table 2). The mucosal inflammation was statistically correlated to *H. pylori* colonization (Table 3, X^2^ = 102.68, *p* <  0.001). In young group, the antrum presented more severe inflammation with *H. pylori* infection (*p* < 0.001), comparing with IA and corpus. However, the mucosal inflammation density in corpus dramatically increased in elderly group (*p* < 0.001) and did not show the significant difference with antrum in the same age group. These data demonstrate that the *H. pylori* associated mucosal inflammation aggravated in elderly patient, especially in proximal stomach.

**Table 2:** The characters of *H. pylori* associated gastric mucosal inflammation in different age population: *H. pylori* associated mucosal inflammation aggravate in elderly patient, especially in proximate stomach

Age group	Antrum	IA	Corpus	*p* Value
Young group(20y~25y)	1.20 ± 0.17	0.85 ± 0.13	0.37 ± 0.10	< 0.001
Mid-aged group(40y~50y)	1.57 ± 0.19	1.45 ± 0.19	1.03 ± 0.13	0.077
Elderly group(>60y)	1.72 ± 0.19	1.77 ± 0.17	1.83 ± 0.18	0.902
Kruskal-Wallis H (*p*)	0.132	< 0.001	< 0.001	–

*IA* Incisura angularis, Data present as standard error of the mean

**Table 3:** The characters of *H. pylori* associated gastric mucosal inflammation in different age population: The mucosa inflammation is consistent with the severity of *H. pylori* colonization

Mucosal inflammation	*H.pylo*ri(+)	*H.pylori* (−)	Total
+	292	30	322
–	114	104	218
Total	406	134	540

X^2^ = 102.68, *p* < 0.001

### Pyloric metaplasia frequently present in elderly population

**Fig. 3:**
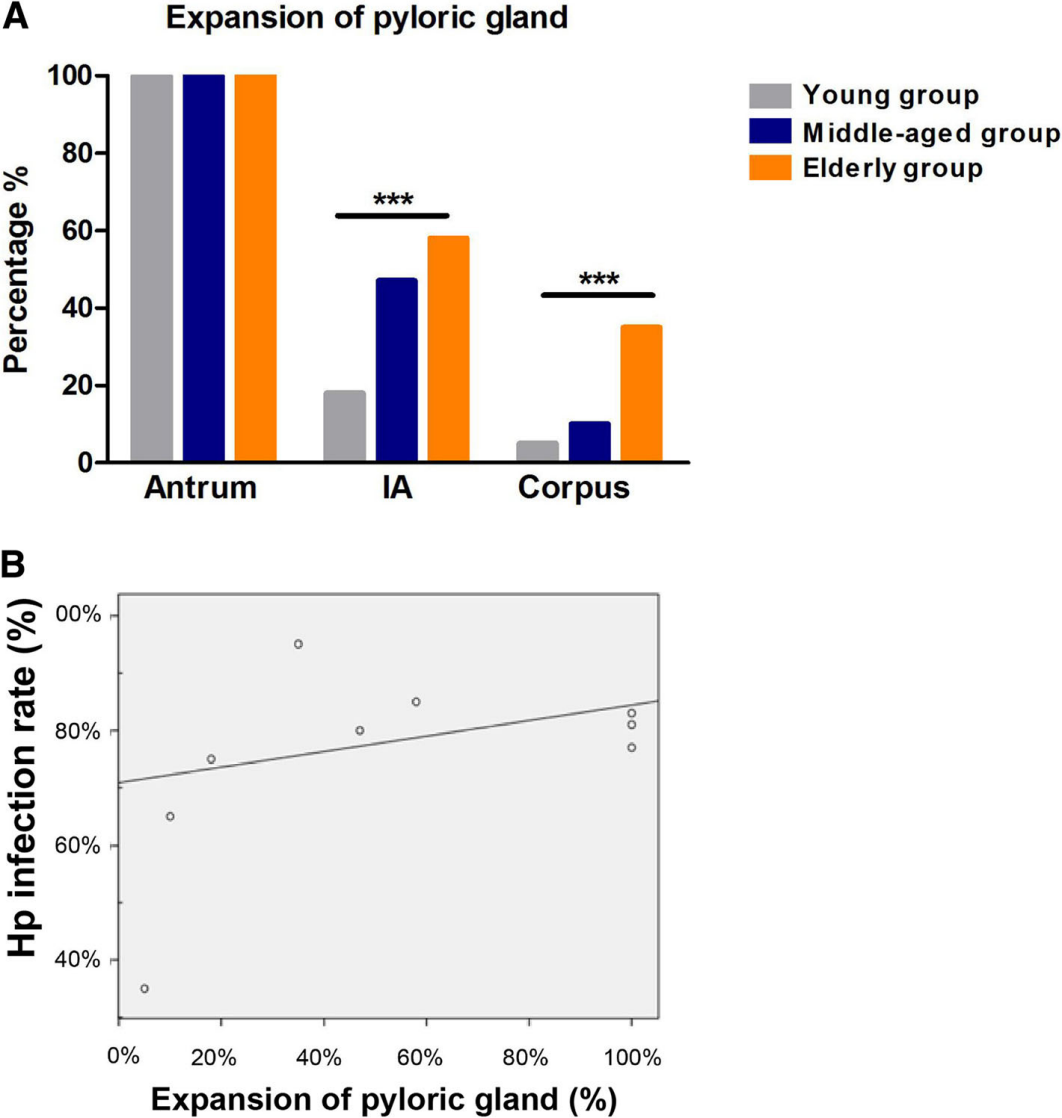
The association of *H. pylori* distribution and pyloric metaplasia with increasing age. **a** Distribution of pylori gland in different sites of stomach in different age groups. *** *p* < 0.001. **b** The correlation between the infection rate of *H. pylori* and the gland type expansion in five sites among three age groups

Among three different age groups, the detection rate of pyloric gland present equally 100% in antrum, 18%, 47%, 58% respectively in IA and 5%, 10%, 35% in corpus, shown as Fig. 3a. The frequencies of pyloric gland in IA and corpus were significantly increased with increasing age (Fig. 3a, X^2^ = 20.97, *p* < 0.001; X^2^ = 22.32, *p* < 0.001).

### The colonization of *H. pylori* and *H. pylori* associated mucosal inflammation are correlated to the gland type expansion with age separately

The Pearson correlation was used to observe the relation between *H. pylori* infection rate and proximal expansion of pyloric gland. The coefficient was 0.804 (Fig. 3b, *p* < 0.01). The data showed that *H. pylori* infection dynamically correlated with pyloric gland with increasing age.

The correlation of *H. pylori* associated mucosal inflammation and pyloric metaplasia was determined with Chi-Square Goodness-of-Fit Test. The mucosal inflammation presents a correlation with pyloric metaplasia similar as *H. pylori* infection with age-dependent tendency (Table 4, X^2^ = 106.14, *p* < 0.001).

**Table 4:** The correlation between the colonization of *H. pylori*-associated mucosal inflammation and the gland type expansion

Mucosal inflammation	*Pyloric gland* (+)	*Fundic gland* (−)	Total
+	228	94	322
–	56	162	218
Total	284	256	540

X^2^ = 106.14, *p* < 0.001

### Pyloric metaplasia in corpus is correlated with the aggravated *H. pylori* colonization and associated inflammation in elderly population

As shown above, the *H. pylori* infection rates, severity of *H. pylori* colonization and mucosal inflammation predominantly aggravated in corpus with increasing age, comparing with antrum and IA. The increase of pyloric metaplasia in corpus with age was also confirmed as Fig. 3a depicted. To evaluate the mechanism of pyloric metaplasia with age on *H. pylori* associated inflammation in corpus, the correlation among *H. pylori* colonization, severity of mucosal inflammation, pyloric metaplasia and age was further measured with zero-order (Pearson) multiple correlations. Mucosa inflammation and *H. pylori* density were evaluated as variables in the calculation with pyloric gland and age as controlling variables. The Table 5 revealed the significant correlation among these four dimensions (*p* < 0.001), which indicated that pyloric metaplasia in corpus was correlated with the aggravated *H. pylori* colonization and inflammation in elderly population.

**Table 5:** Multiple correlation analysis among mucosal inflammation, *Hp* density, the pyloric gland and age in corpus

Correlations^a,b,c^		Mucosal inflammation	*Hp* density	Pyloric gland	Age
Mucosal inflammation	Correlation	1.00	0.90	0.57	0.48
	Significance(2-detailed)	–	0.00	0.00	0.00
*Hp* density	Correlation	0.90	1.00	0.43	0.50
	Significance(2-detailed)	0.00	–	0.00	0.00
Pyloric gland	Correlation	0.57	0.43	1.00	0.33
	Significance(2-detailed)	0.00	0.00	–	0.00
Age	Correlation	0.48	0.50	0.33	1.00
	Significance(2-detailed)	0.00	0.00	0.00	–

^a^cells contain zero-order (Pearson) correlations, ^b^mucosal inflammation and *Hp* density were choosen as variables, ^c^pyloric gland and age were set as controlling variables

## Discussion

Epidemiologic studies on *H. pylori* infection in elderly people reported a prevalence of 60% in asymptomatic subjects and more than 70% in elderly patients with gastrointestinal diseases [25]. In particularly, the elderly population presents a significantly higher mean morbidity of *H. pylori* associated diseases [2–6], such as chronic gastritis, gastric ulcer and gastric carcinoma, especially in proximal stomach [11, 12, 15]. However, the mechanisms underlying the markedly elevated morbidity of *H. pylori* associated diseases in proximal stomach of elderly population are still not clear.

In the present study, biopsies from five predetermined sites in stomach were taken from each patient to approach the correlation among *H. pylori* distribution, mucosa inflammation, gland type and age in different areas of stomach. Misra et al. have shown the utility of using the same biopsy specimen for two tests [26].

The average infection rate of *H. pylori* in antrum and gastric corpus were about 80% and 65% respectively in our study, which were higher than developed countries [25, 27, 28]. The subjects observed in present study are patients with dyspepsia symptoms,which might be the reason for the higher *H. pylori* infection rate. It was also probably due to public health condition and eating habit in China. Note, data from our research further demonstrated that the aggravations of both *H. pylori* infection rate and *H. pylori* colonization severity in corpus was more conspicuous than the antrum and IA with increasing age, which clearly revealed that the distribution of *H. pylori* extends to proximal stomach with increasing age.

The mucosal inflammation was also observed in functional gastrointestinal disorder patients with dyspepsia symptom. The severity of mucosa inflammation was consistent with *H. pylori* colonization among three age groups, and presents a distal-to-proximal gastric direction similar as *H. pylori* infection with increasing age.

The pyloric metaplasia in corpus was significantly upgraded in elderly population compared with young group and middle-aged group, which revealed that pyloric metaplasia tends to proximal stomach with increasing age. This age-related tendency of pyloric gland running to proximal stomach has also been reported by Van Zanten et al. [15, 17].

We further compared the correlation of the colonization of *H. pylori*, mucosal inflammation and distribution of pyloric gland in different stomach sites among three age groups. A statistically significant positive correlation was obtained. The data revealed that pyloric metaplasia in corpus was correlated with the aggravated *H. pylori* colonization and associated inflammation in elderly population.

As described in the introduction, pyloric gland and fundus gland in stomach present different cell types, the different pathophysiologic characters on pH levels [17], mucin glycoforms [18], binding manner of *H. pylori* [19]. *H. pylori* have been shown prefer to colonize first and initiate in the lower part of stomach (pyloric gland). Therefore, the running tendency of antral-corpus transitional zones with age might contribute to the high morbidity of *H. pylori* in proximal stomach of elderly population.

Moreover, *H. pylori* infection could aggravate mucosa atrophy. The parietal cells and chief cells of fundic glands can be partially insteaded by mucus cells of pylori glands after the *H. pylori* infection [2, 15, 29, 30], which might accelerate transitional zones moving to proximal of stomach. Besides, antrum-corpus transitional zones were known as a sanctuary site in eradication failure [31].

## Conclusions

Based on the findings in the present study, it is concluded that both *H. pylori* distribution and mucosal inflammation present a tendency running to the proximal of stomach with increasing age, which correlated with pyloric metaplasia. We speculated this might make a reasonable explanation for the higher morbidity of *H. pylori* associated diseases in proximal stomach of elderly population.

The limitation of this study is that there is the potential bias due to time-cohort effect with different environmental factors exposed among different age groups, but the positive association among *H. pylori* colonization and pyloric metaplasia is demonstrated within each age group. Further clinical trials evaluating the effect of eradication of *H. pylori* infection on the pyloric metaplasia process are now needed to further understand.

## Abbreviations

CLE: Confocal laser endomicroscopy
IA: Incisura angularis
URUT: Ultra-rapid urease test
